# Use of ticagrelor and the risks of pneumonia and pneumonia-specific death in patients with non-acute coronary syndrome conditions: a population-based cohort study

**DOI:** 10.1038/s41598-021-00105-z

**Published:** 2021-10-14

**Authors:** Qi Feng, Man Fung Tsoi, Yue Fei, Ching Lung Cheung, Bernard M. Y. Cheung

**Affiliations:** 1 Division of Clinical Pharmacology and Therapeutics, Department of Medicine, Faculty of Medicine, The University of Hong Kong, Hong Kong, China; 2 Department of Pharmacology and Pharmacy, LKS Faculty of Medicine, Queen Mary Hospital, The University of Hong Kong, Hong Kong, China; 3 State Key Laboratory of Pharmaceutical Biotechnology, The University of Hong Kong, Hong Kong, China; 4 Institute of Cardiovascular Science and Medicine, The University of Hong Kong, Hong Kong, China

**Keywords:** Cardiology, Medical research

## Abstract

Previous studies have shown that ticagrelor reduced risk of pneumonia in patients with acute coronary syndrome (ACS) compared to clopidogrel, however, its effect in patients with non-ACS cardiovascular diseases remains uncertain. The aim was to investigate the effect of ticagrelor on pneumonia and pneumonia-specific death compared to clopidogrel in non-ACS patients in Hong Kong. This was a population-based cohort study. We included consecutive patients using ticagrelor or clopidogrel admitted for non-ACS conditions in Hong Kong public hospitals from March 2012 to September 2019. Patients using both drugs were excluded. The outcomes of interest were incident pneumonia, all-cause death, and pneumonia-specific death. Multivariable survival analysis models were used to estimate the effects [hazard ratio (HR) and 95% confidence interval (CI)]. Propensity score matching, adjustment and weighting were performed as sensitivity analyses. In total, 90,154 patients were included (mean age 70.66 years, males 61.7%). The majority of them (97.2%) used clopidogrel. Ticagrelor was associated with a lower risk of incident pneumonia [0.59 (0.46–0.75)], all-cause death [0.83 (0.73–0.93)] and pneumonia-specific death [0.49 (0.36–0.67)]. Sensitivity analyses yielded similar results. Ticagrelor was associated with lower risk of all-cause death, pneumonia-specific death, and incident pneumonia in patients with non-ACS cardiovascular conditions, consistent with previous evidence in patients with ACS. This additional effect of anti-pneumonia should be considered when choosing a proper P2Y12 inhibitor for patients with high risk of pneumonia.

## Introduction

Cardiovascular diseases (CVDs) are one of the major contributors to global disease burden, especially in aging populations^1,2^. P2Y12 inhibitors, including clopidogrel and ticagrelor, are effective antiplatelet drugs for CVD treatment^3^. Ticagrelor is recommended over clopidogrel to patients with acute coronary syndrome (ACS) after percutaneous coronary intervention or coronary artery bypass grafting to improve cardiovascular outcomes^4–6^. In addition to ACS, P2Y12 inhibitors have been used to prevent atherothrombosis in patients with other cardiovascular conditions, such as peripheral vascular diseases and stable ischemic heart disease^7,8^. The Effect of Ticagrelor on Health Outcomes in Diabetes Mellitus Patients Intervention Study (THEMIS) has shown its protective effects in patients with stable coronary artery disease on cardiovascular death, myocardial infarction and stroke, although the increased risk of bleeding decreased its net clinical benefit^9^.

P2Y12 inhibitors are generally believed to increase the risk of infection. For example, clopidogrel is associated with an increased risk of infection by 48%^10^ to 51%^11^ compared to placebo, which is supported by in vitro studies^12^. However, studies have suggested that ticagrelor has the potential to reduce the risk of pneumonia, in contrast to clopidogrel. Recent in vivo and in vitro studies have revealed its antibacterial effects against Gram-positive bacteria^13,14^. The Platelet Inhibition and Patient Outcomes (PLATO) trial found that ACS patients treated with ticagrelor had lower risks of pneumonia and infection-related death than clopidogrel^15,16^. The same trend was also observed in patients with peripheral artery disease in the Examining Use of Ticagrelor in Peripheral Artery Disease (EUCLID) trial, albeit insignificant^17^. A recent meta-analysis^18^, combining 10 randomized controlled trials, showed that ticagrelor significantly reduced the risk of pneumonia by 20%, but had null effect on upper respiratory tract infection, urinary tract infection, or sepsis. The review^18^ demonstrated that the risk reduction of pneumonia was significant in patients with ACS, but remained inconclusive in patients with non-ACS conditions, possibly due to small sample size and low statistical power, which merits more investigations.

On the other hand, the generalizability of these findings remains unknown. Most of the current evidence was generated from the Caucasian population; for example, 99.5% of total patients were Caucasian in that meta-analysis^18^. All the evidence was generated from well-controlled randomized trials with carefully selected patients, which may limit their generalizability to real-world settings. Therefore, the objective of this study was to examine the effect of ticagrelor compared to clopidogrel on the risk of pneumonia and related death in patients with conditions other than recent ACS in a population-based cohort of Hong Kong Chinese. The research hypothesis was that ticagrelor users had lower risk of pneumonia and pneumonia-specific death, compared to clopidogrel users.

## Results

**Figure 1:**
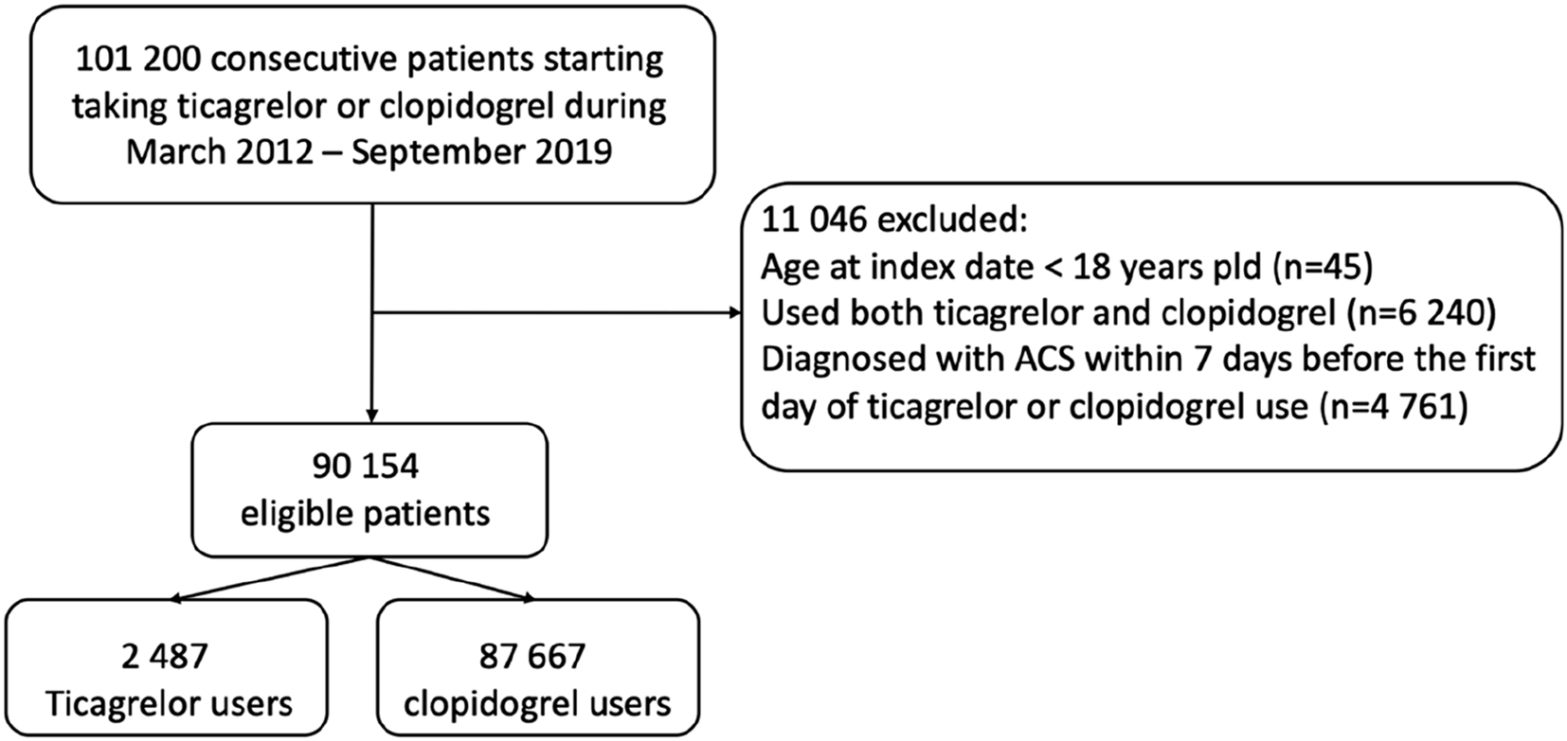
Flowchart for patient selection.

In total, 90,154 patients (representing 269,773 person-years of follow-up) were included (Fig. 1). Their characteristics are shown in Table 1. The mean age was 70.66 years and 61.7% were males. Among all eligible patients, 2487 (2.8%) were ticagrelor users. The reasons for patients receiving ticagrelor or clopidogrel included: history of ACS (22%), stable coronary artery disease (15%), stroke (20%), peripheral artery diseases (5%) and other reasons, such as more than one indication, indication not stated, research, private prescription, intolerance of other antiplatelet drugs, etc. Ticagrelor was administered at a dose of 90 mg twice daily, while clopidogrel at a dose of 75 mg daily. Compared with clopidogrel users, ticagrelor users were more likely to be male, younger, and to have lower Charlson comorbidity index and shorter drug duration. Ticagrelor users had fewer hospital admissions but more outpatient visits during the 12 months before their treatment initiation date (Table 1).

**Table 1:** Baseline characteristics of the patients with conditions other than recent ACS.

	Ticagrelor users	All clopidogrel users	PS-matched clopidogrel users
Sample size	2487	87,667	7461
Male	1964 (78.97)	53,668 (61.22)	5920 (79.35)
Age	62.47 (11.49)	70.09 (13.47)	62.16 (12.82)
Charlson comorbidity index 1–2	310 (12.46)	16,434 (18.75)	945 (12.67)
Median follow-up time (months)	23.40	31.70	22.70
Mean drug duration (days)	285.20	316.90	279.26
Median number of hospitalizations	1	1	1
Mean number of hospitalizations	1.48	2.67	1.67
Median number of outpatient visits	4	2	2
Mean number of outpatient visits	5.43	4.67	5.03

*ACS* acute coronary syndrome, *PS* propensity score.

The median follow-up lengths were 23.40 and 31.70 months for ticagrelor users and clopidogrel users, during which 265 (10.66%) and 23 038 (26.27%) deaths were recorded, respectively. In multivariable Cox model, ticagrelor significantly reduced all-cause mortality by 17% [hazard ratio (HR) 0.83 (95% confidence interval 0.73–0.93)]. There were 6807 deaths due to pneumonia (39 in ticagrelor group and 6768 in clopidogrel group). Ticagrelor users showed a reduced risk of pneumonia-specific death by 51% [HR 0.49 (0.36–0.67)], which was consistent with the results of Fine-Gray model that takes into account of competing causes of death [HR 0.47 (0.34–0.67)]. 8524 pneumonia cases were captured (61 in ticagrelor users and 8460 in clopidogrel users) during follow-up. Use of ticagrelor was associated with a reduced risk of incident pneumonia by 41% [HR 0.59 (0.41–0.75)] (Table 2).

**Table 2:** The association between use of ticagrelor with pneumonia-specific death, all-cause death, and incident pneumonia in the overall cohort.

		Ticagrelor (event/total)	Clopidogrel (event/total)	HR (95% CI)
Pneumonia-specific death	Cox model	39/2487	6768/87,667	0.49 (0.36, 0.67)
	Fine-Gray model	39/2487	6768/87,667	0.47 (0.34, 0.67)
All-cause death	Cox model	265/2487	23,038/87,667	0.83 (0.73, 0.93)
Incident pneumonia	Cox model	64/2487	8460/87,667	0.59 (0.46, 0.75)

Adjusted for age, gender, Charlson Comorbidity Index, number of outpatient visits during previous 12 months, number of hospitalizations during previous 12 months, year of first drug dispensing date, duration of drug use, baseline cerebrovascular diseases, diabetes and chronic obstructive pulmonary disease. Fine-Gray model is a survival model taking into account competing risk.

In propensity score matching analysis, 2487 ticagrelor users and 7461 clopidogrel users were included. The two matched groups showed balanced baseline characteristics (Table 1). Significant effects on pneumonia-specific death [0.55 (0.39–0.78)] and incident pneumonia [0.65 (0.49–0.85)] were observed. The effect of ticagrelor on all-cause death [1.01 (0.88–1.16)] became insignificant, possibly due to reduced sample size. Propensity score adjustment in multivariable regression and inverse probability treatment weighting analysis also yielded similar results (Table 3).

**Table 3:** The association between use of ticagrelor with pneumonia-specific death, all-cause death, and incident pneumonia, using propensity-score matching, adjustment and weighting methods.

		Ticagrelor (event/total)	Clopidogrel (event/total)	HR (95% CI)
**Propensity-score matching**				
Pneumonia-specific death	Cox model	39/2487	213/7461	0.55 (0.39, 0.78)
	Fine-Gray model	39/2487	213/7461	0.55 (0.39, 0.77)
All-cause death	Cox model	265/2487	868/7461	1.01 (0.88, 1.16)
Incident pneumonia	Cox model	64/2487	305/7461	0.65 (0.49, 0.85)
**Propensity-score adjustment**				
Pneumonia-specific death	Cox model	39/2487	6768/87,667	0.56 (0.41, 0.77)
	Fine-Gray model	39/2487	6768/87,667	0.51 (0.40, 0.70)
All-cause death	Cox model	265/2487	23,038/87,667	0.94 (0.83, 1.06)
Incident pneumonia	Cox model	64/2487	8460/87,667	0.67 (0.53, 0.86)
**Inverse probability treatment weighting**				
Pneumonia-specific death	Cox model	39/2487	6768/87,667	0.49 (0.33, 0.74)
	Fine-Gray model	39/2487	6768/87,667	0.47 (0.34, 0.67)
All-cause death	Cox model	265/2487	23,038/87,667	0.22 (0.10, 0.49)
Incident pneumonia	Cox model	64/2487	8460/87,667	0.64 (0.48, 0.86)

Adjusted for age, gender, Charlson Comorbidity Index, number of outpatient visits during previous 12 months, number of hospitalizations during previous 12 months, year of first drug dispensing date, duration of drug use, baseline cerebrovascular diseases, diabetes and chronic obstructive pulmonary disease. Fine-Gray model is a survival model taking into account competing risk.

## Discussion

The main findings of this population-based cohort study are that, compared to clopidogrel, use of ticagrelor was associated with lower risk of all-cause death, incident pneumonia, and pneumonia-specific death in patients with non-ACS conditions, similar to the pneumonia-protective effect previously observed in ACS patients^18^.

We observed that ticagrelor was associated with a risk reduction in overall mortality. A meta-analysis^19^ of 12 randomized controlled trials found a similar risk reduction in ACS patients. Among non-ACS patients, previous evidence remains inconsistent. In the LEADER trial^20^, ticagrelor reduced all-cause mortality in patients with stable coronary artery diseases, which is similar to our finding. By contrast, Chen et al*.*^21^ found a null association, which might be resulted from the limited sample size (n = 344) and heterogeneity in patient characteristics. One of the potential mechanisms for the protective effect on all-cause mortality is that the ticagrelor, as a reversible P2Y12 inhibitors, allows quicker restoration of platelet function^19^. In the PLATO trial, although ticagrelor did not decrease risk of major bleeding, it did not increase the risk of procedure-related bleeding. By contrast, ticagrelor increased risk of non-procedure-related bleeding, including gastrointestinal bleeding, which was less fatal than procedure-related bleeding. The reduced rate of fatal bleeding, together with reduced mortality from vascular causes and other causes contributed to the lower mortality in ticagrelor group of ACS patients^22^. However, more research is warranted to confirm the association and delineate the mechanisms in non-ACS patients.

Previous evidence regarding the effect of ticagrelor on pneumonia among non-ACS people has been inconclusive. The EUCLID study^17^ and Steblovnik et al.^23^ observed similarly insignificant risk reduction of pneumonia in ticagrelor users among patients with peripheral artery disease and cardiac arrest, respectively; nevertheless, their results may be limited by low statistical power and small numbers of pneumonia cases during follow-up (301 in the EUCLID study^17^ and 15 in Steblovnik et al.^23^). Whereas in the present study, more cases of incident pneumonia (n = 8524) have been accumulated in a large sample size during follow-up, which lent high statistical power to our analysis. The finding that patients treated with ticagrelor had a statistically significant lower risk of pneumonia, was consistent with the results of sensitivity analyses, which further reinforces the internal validity.

Two potential mechanisms have been proposed for ticagrelor’s protective effect on pneumonia-associated outcomes. First, ticagrelor can increase extracellular adenosine, via inhibiting the ENT-1 receptor to restrict cellular uptake of adenosine^24^. Adenosine has been shown to activate neutrophils to release cytokines and chemokines, to promote phagocytosis, neutrophil chemotaxis and degranulation via low-affinity G protein-coupled receptors, as well as resolution of lung injury^25–27^. Second, in vivo and in vitro studies have revealed the direct antimicrobial effect of ticagrelor, especially on Gram-positive bacteria strains^13,14^. Ticagrelor has shown promising role in preventing multi-organ failure in patient with sepsis due to resistant Gram-positive *cocci*^28^. However, when interpreting the results, we can not exclude the possibility that the differential effects between ticagrelor and clopidogrel was due to clopidogrel-associated increased infection risk, more than ticagrelor’s protective effect. A further analysis of inflammatory biomarkers in the PLATO trial suggested an immunosuppressive effect of clopidogrel on reducing white blood cell count, which was independent of baseline biomarkers and clinical risk factors^29^. By contrast, ticagrelor is associated with higher levels of inflammatory biomarkers, such as C-reactive protein and interleukin-6^15^. This difference (clopidogrel’s immunosuppressive effect versus ticagrelor’s pro-inflammatory and antimicrobial effect) may play important roles in sepsis, especially in the hypo-inflammatory phase in sepsis, which is more lethal than the initial hyper-inflammatory phase^30,31^, thus contributing to the lower sepsis-specific mortality of ticagrelor.

In this study, patients treated with ticagrelor showed lower risk of pneumonia-specific mortality. Among ACS patients, the PLATO trial^16^ revealed a significant reduction in death risk due to any infection, and that ticagrelor was associated with lower mortality following pulmonary adverse events (including infection and non-infection)^15^. However, this trial did not show direct evidence on pneumonia-specific mortality so far. Previous investigation on this outcome in non-ACS patients is very limited, and the present study serves as an important contribution to the evidence pool. Among patients with pneumonia, use of ticagrelor reduced platelet-leukocyte interaction, lowered interleukin-6 level, and improved lung function^32^, serving as potential physiological mechanisms how it prevents pneumonia-specific death.

The cardiovascular benefits of ticagrelor have been well evidenced^4,19^. In this study, we demonstrated its additional benefits in reducing risks of pneumonia-associated outcomes in patients with non-ACS conditions, consistent with the pneumonia-protective effect previously observed in ACS patients^18^. This benefit should be taken into consideration when prescribing P2Y12 inhibitor to patients with high risk of pneumonia, such as the elderly, smokers, COPD patients, and the immunosuppressed.

We acknowledge some limitations of this study. First, this study included imbalanced numbers of ticagrelor- and clopidogrel-users, which might cause statistical instability, but propensity-score matching analysis (having comparable numbers in the two groups) generated similar results on pneumonia outcomes to the primary analysis. Second, we did not take the drug dosage into account, which may vary across medical conditions and temporally during the long follow-up period. Third, we did not examine potential drug interactions in this study. The population with a mean age of 70 years old usually take multiple medications^33^, such as antihypertensives and statins, but it remains unclear whether these medications interact with ticagrelor or clopidogrel, and whether these interactions would affect pneumonia-related outcomes, if any. Fourth, this is an observational study, thus residual confounding effect may still exist even when we have adjusted for multiple baseline characteristics. Fifth, we did not assess the baseline pneumonia risk and did not include pneumonia-associated risk factors in analysis, due to lack of relevant data.

## Conclusion

In this large population-based cohort study of 90,154 patients without ACS, use of ticagrelor was associated with lower risk of all-cause death, pneumonia-specific death, and incident pneumonia. This additional effect against pneumonia should be taken into consideration when choosing P2Y12 inhibitors for patients with high risk of pneumonia.

## Methods

### Population and settings

This was a population-based retrospective cohort study, designed within the Hong Kong Clinical Data Analysis and Reporting System (CDARS), a database initiated and managed by the Hong Kong Hospital Authority. CDARS contains individual patient information from all local public hospitals in Hong Kong on their demographics, diagnoses, laboratory tests, prescription and treatment. The database captures more than 90% of the Hong Kong population, and serves as an excellent data source for population-based observational research. More information about the CDARS and its use in clinical research has been reported previously^34^. All information extracted for research purpose was anonymous and unidentifiable, thus written informed consents were waived by the University of Hong Kong & Hospital Authority Hong Kong West Cluster Institutional Review Board. This study received ethical approval from the University of Hong Kong & Hospital Authority Hong Kong West Cluster Institutional Review Board (IRB No. UW20-153). All research was performed in accordance with the approved protocol, guidelines and regulations.

### Data collection and variable measurement

We obtained data of the patients who have used ticagrelor or clopidogrel from CDARS. Patients were included if the date of their first use of ticagrelor or clopidogrel was between March 2012 and September 2019, or if they did not have diagnosis of ACS within seven days before the first day of ticagrelor or clopidogrel use. March 2012 was the time when ticagrelor was approved in Hong Kong, before which clopidogrel was the only option. Diagnosis of ACS was identified with the International Classification of Diseases (ICD)-9 code 410, 411.1, and 411.8. Patients were excluded if: (1) they did not collect their prescribed P2Y12 inhibitors, (2) they were < 18 years old at their first exposure to ticagrelor or clopidogrel, (3) they had previous exposure to ticagrelor or clopidogrel before March 2012, and (4) they used ticagrelor and clopidogrel simultaneously (n = 828), or they switched between the P2Y12 inhibitors during follow-up, either from ticagrelor to clopidogrel (n = 822), or from clopidogrel to ticagrelor (n = 4590). We included all consecutive eligible patients.

The exposure was use of ticagrelor, and clopidogrel users were the control. The index date was the date of first exposure to ticagrelor or clopidogrel. The outcomes of interest were all-cause death, pneumonia-specific death, and incident pneumonia. Pneumonia-specific death was defined by ICD-10 code J12–J18. Incident pneumonia included pneumonia-specific death and/or any pneumonia diagnosis made after treatment initiation dates (identified by ICD-9 code 480–486), whichever occurred first. For patients with multiple diagnoses of pneumonia, we included the first confirmed diagnosis. Outcome validation was done by reviewing the medical records of a random sample of 200 patients, which yielded both positive predictive value and negative predictive value of 100%.

Covariates included age, gender, baseline comorbidity index, number of hospital admissions during the past 12 months before the index date, number of outpatient visits during the past 12 months, year of the index date, duration of drug use, and baseline cerebrovascular disease, diabetes, chronic obstructive pulmonary disease. Overall baseline comorbidity status was measured with Charlson comorbidity index^35^, based on all relevant confirmed diagnoses before index date. Baseline cerebrovascular disease, diabetes, and chronic obstructive pulmonary disease were specifically measured. Duration of drug use (in days) was defined as the sum of all non-overlapping durations specified in prescription notes.

### Statistical analysis

The proportion of missing data was negligible in CDARS (< 1.00%), thus complete-case analysis was used. Baseline characteristics were summarized with descriptive statistics and compared between ticagrelor users and clopidogrel users. Continuous variables were summarized using mean with standard deviation or median with interquartile range, while binary and categorical variables were summarized using frequency with proportion.

Survival analysis was used to evaluate the association between ticagrelor use and the outcomes. The effect sizes between ticagrelor use and the health outcomes were estimated with Cox proportional hazard model, adjusted for age, gender, Charlson comorbidity index, the number of outpatient visits during the previous 12 months, the number of hospital admissions during the previous 12 months, year of index date, duration of drug use, and baseline cerebrovascular disease, diabetes, and chronic obstructive pulmonary disease. The assumption of hazard proportionality in Cox model was examined with scaled Schoenfeld residuals, and no evidence of violation was observed in analysis. For pneumonia-specific death, Fine-Gray model was additionally fitted to deal with competing risks^36,37^, adjusting for the same covariates.

For sensitivity analyses, we employed propensity score approach to examine the robustness of the results. The propensity score was the probability of receiving ticagrelor, calculated from a logistic regression model with ticagrelor use as the dependent variable and all other baseline covariates as independent variables. Propensity score matching, adjustment and inverse probability of treatment weighting (IPTW) were performed. Matching with a ratio of 1:3 (= ticagrelor: clopidogrel) was employed. In propensity score adjustment analysis, the logit of the propensity score was fitted with a restricted cubic spline function to adjust for any potential non-linear effect. In IPTW analysis, the weight was computed as 1/propensity score for ticagrelor users and 1/(1 − propensity score) for clopidogrel-users.

Effect size was expressed as hazard ratio (HR) and its 95% confidence interval (CI), with an HR > 1 suggesting an increased risk of outcome with the use of ticagrelor. Statistical significance level was specified at 0.05. Data analysis was conducted with “*rms*” “*cmprsk*” and “*MatchIt*” packages in R environment (R core team, 2013).

### Ethical approval

This study received ethical approval from the HKU/HA HKW Institutional Review Board (IRB No. UW 20-153).
